# Association of AI-determined Kellgren–Lawrence grade with medial meniscus extrusion and cartilage thickness by AI-based 3D MRI analysis in early knee osteoarthritis

**DOI:** 10.1038/s41598-023-46953-9

**Published:** 2023-11-16

**Authors:** Ichiro Sekiya, Hisako Katano, Ali Guermazi, Yugo Miura, Noriya Okanouchi, Makoto Tomita, Jun Masumoto, Yoshio Kitazume, Hideyuki Koga, Nobutake Ozeki

**Affiliations:** 1 Center for Stem Cell and Regenerative Medicine, Tokyo Medical and Dental University, Tokyo, Japan; 2 Quantitative Imaging Center, Department of Radiology, Boston University School of Medicine, Boston, MA USA; 3 School of Data Science, Graduate School of Data Science, Yokohama City University, Kanagawa, Japan; 4 Fujifilm Corporation, Tokyo, Japan; 5 Department of Diagnostic Radiology and Nuclear Medicine, Tokyo Medical and Dental University, Tokyo, Japan; 6 Department of Joint Surgery and Sports Medicine, Graduate School of Medical and Dental Sciences, Tokyo Medical and Dental University, Tokyo, Japan; 7 Center for Stem Cell and Regenerative Medicine, Tokyo Medical and Dental University (TMDU), 1-5-45 Yushima, Bunkyo-ku, Tokyo, 113-8510 Japan

**Keywords:** Cartilage, Osteoarthritis

## Abstract

The associations among Kellgren–Lawrence (KL) grade, medial meniscus extrusion (MME), and cartilage thickness in knee osteoarthritis (OA) remain insufficiently understood. Our aim was to determine these associations in early to moderate medial tibiofemoral knee OA. We included 469 subjects with no lateral OA from the Kanagawa Knee Study. KL grade was assessed using artificial intelligence (AI) software. The MME was measured by MRI, and the cartilage thickness was evaluated in 18 subregions of the medial femorotibial joint by another AI system. The median MME width was 1.4 mm in KL0, 1.5 mm in KL1, 2.4 mm in KL2, and 6.0 mm in KL3. Cartilage thinning in the medial femur occurred in the anterior central subregion in KL1, expanded inwardly in KL2, and further expanded in KL3. Cartilage thinning in the medial tibia occurred in the anterior and middle external subregions in KL1, expanded into the anterior and middle central subregions in KL2, and further expanded in KL3. The absolute correlation coefficient between MME width and cartilage thickness increased as the KL grade increased in some subregions. This study provides novel insights into the early stages of knee OA and potentially has implications for the development of early intervention strategies.

## Introduction

Osteoarthritis (OA) of the knee is a serious disease, and the aging of the global population is increasing the number of patients affected by this disease. OA is a disabling disease that reduces the ability to walk, mainly due to knee pain^1^. Diagnosis is clinical, and confirmation is based on radiographs, with Kellgren–Lawrence (KL) grading being the most widely used system for OA severity grading/assessment^2^. However, the KL grading system is subject to inter-rater and intra-rater variability due to its reliance on subjective evaluation, which can lead to inconsistencies in repeatability^3^. An artificial intelligence (AI)-based KL labeling system has been developed to mitigate these variabilities, aiming to standardize grading and improve the consistency of assessments^4,5^.

**Figure 1:**
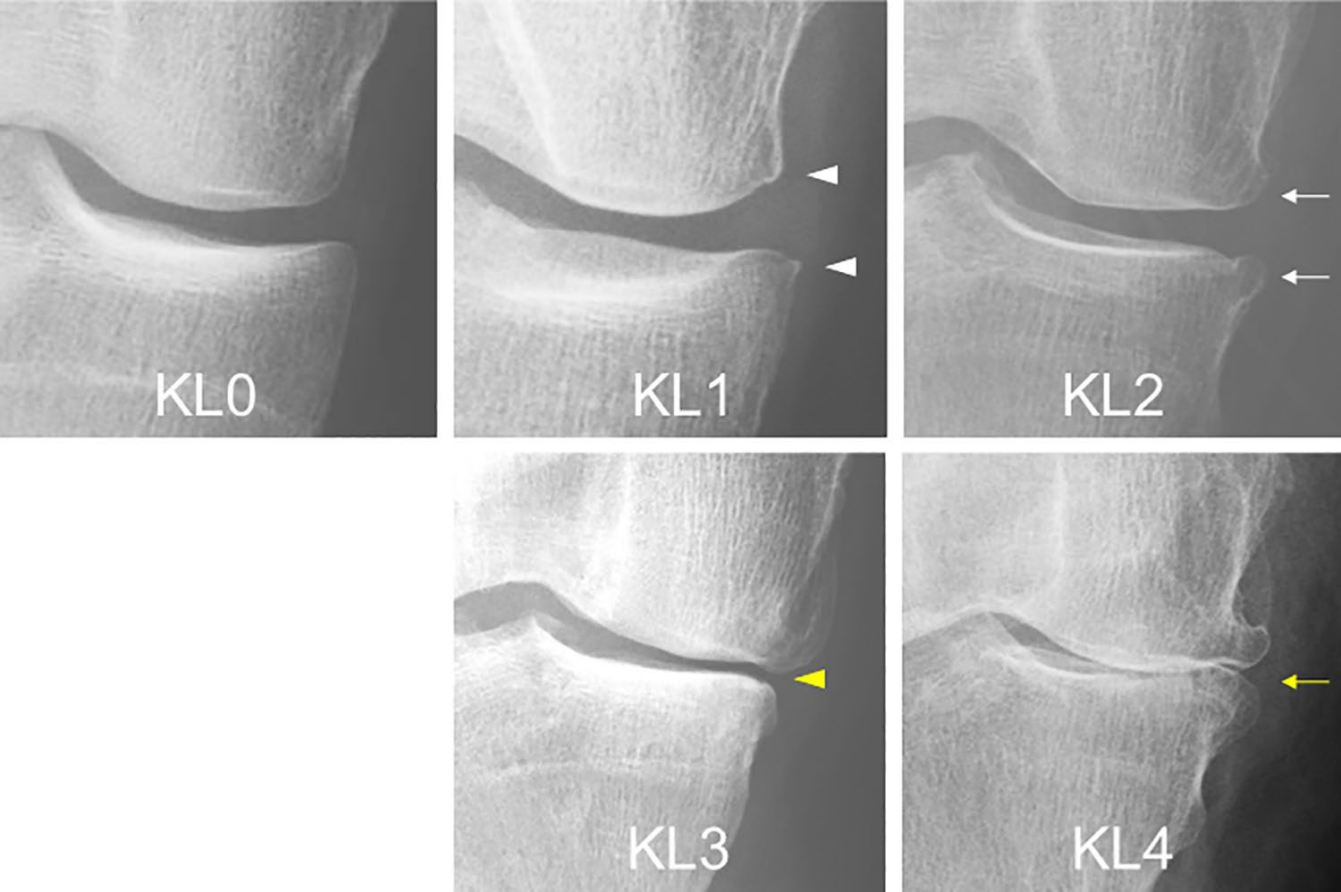
Representative radiographs of KL grading in medial OA. KL grade 0 shows no features of OA. KL grade 1 shows osteophytic lipping (equivocal osteophytes) (white arrow heads). KL grade 2 shows definite (unequivocal) osteophytes (white arrows). KL grade 3 shows joint space narrowing (yellow arrow head). KL grade 4 shows definite deformity of the bone ends (bone-to-bone appearance) (yellow arrow).

The KL grading is based on the number of osteophytes and the joint space narrowing (Fig. 1)^6^, and the medial tibiofemoral knee is the most affected compartment with OA. Joint space narrowing is the result of medial meniscus extrusion (MME) and/or articular cartilage damage to the medial femur and tibia. An increasing number of reports have now shown a correlation between osteophyte width and MME in primary non-traumatic OA^7–9^. MRI has enhanced our understanding of joint space narrowing by providing detailed images of soft tissues, such as cartilage and menisci, allowing for deeper investigation of joint space narrowing as an imaging biomarker. Furthermore, contemporary AI-based 3D analysis systems facilitate the precise and efficient quantification of these soft tissues in three dimensions^10–12^. However, the relationship between the KL grade and MME and the areas that show altered cartilage thickness in each KL grade has still not been described.

The KL grade is often used to stratify patients in clinical trials of disease-modifying OA drugs (DMOAD)^13^. In severe knee OA, the knee structures exhibit irreversible degenerative changes, and total knee replacement is the ultimate treatment strategy. Therefore, to prevent OA and to develop conservative therapies, clinicians should preferably target patients with early to moderate disease^14^, as is done in studies of rheumatoid arthritis^15^, Alzheimer’s disease^16^ and other chronic diseases. The purpose of the present study was to determine the relationship between KL grade and MME, the relationship between KL grade and cartilage thickness, and the areas where cartilage thickness is altered in each KL grade in subjects with early to moderate OA. As subjects, we used the cohort in the Kanagawa Knee Study, a cohort of mainly office workers, aged 30 to 79 years, with relatively uniform age and gender differences, and with no consecutive visits to the hospital for more than 3 months for knee disease^8^. Our study findings are important because they provide new insights into OA progression and suggest that MME and reduced cartilage thickness are early signs of OA and can be used to follow disease progression.

## Materials and methods

### Kanagawa Knee Study and subject enrollment

This study was approved by the IRB of Tokyo Medical and Dental University and was conducted in accordance with the relevant guidelines/regulations. Informed consent was obtained from all participants. In addition, this study was performed in accordance with the Declaration of Helsinki.

The subjects were 573 participants in the Kanagawa Knee Study from Kanagawa and Tokyo prefectures. They included 284 (50%) women, and the overall age ranged from 30 to 79 years, with a median age of 54 years^17^. Only the right knee was evaluated in each subject.

Subjects with a history of knee OA, lower extremity trauma, previous surgery, rheumatoid arthritis, or consecutive visits to one or more hospitals for more than 3 months were excluded to remove any treatment influences. Knee radiographs and MRI scans were performed between September 1, 2018, and August 30, 2019. Height and weight were also obtained, and body mass index (BMI) was calculated^9^. Our current study excluded subjects with lateral OA from the Kanagawa Knee Study.

### Automatic evaluation of KL grade

Radiographs were taken of the anteroposterior view of the knee with the patient in a standing position with full knee extension^18^. DICOM data were sent to the Image Biopsy Lab (Vienna, Austria), and KL grading was automatically performed using the Knee Osteoarthritis Labeling Assistant (KOALA) software (IBLAB-ZOO 1.13.16 KOALA-CE)^19^. The grading was reviewed and checked for errors by trained staff at the Image Biopsy Lab; however, to prevent bias, no manual changes were made.

### Exclusion of lateral OA

Parameters for the OARSI grade were also automatically evaluated using the KOALA software: joint space narrowing, medial (JSM); joint space narrowing, lateral (JSL); osteophytes, tibia medial (OSTM); osteophytes, tibia lateral (OSTL); osteophytes, femur medial (OSFM); and osteophytes, femur lateral (OSFL). Each was graded as 0 (normal), 1 (mild change), 2 (moderate change), or 3 (severe change), with greater values indicating a more severe form of OA^20^. To exclude lateral OA, subjects were excluded when the JSL was larger than the JSM and the difference between the OSFL and the OSTL was larger than the difference between the OSFM and the OSTM.

### Magnetic resonance imaging

MRI was performed at 3.0 T (Achieva 3.0TX, Philips, Amsterdam, Netherlands) using a dedicated 16-channel transmit/receive knee coil (dStream T/R Knee 16ch coil, Philips). We acquired 2D sagittal fat-suppressed spoiled gradient echo (SPGR) and proton density-weighted (PDW) sequences (Supplementary Table 1)^9^. The rest time before the MRI scan was approximately 20 min.

### Measurements of cartilage thickness

**Figure 2:**
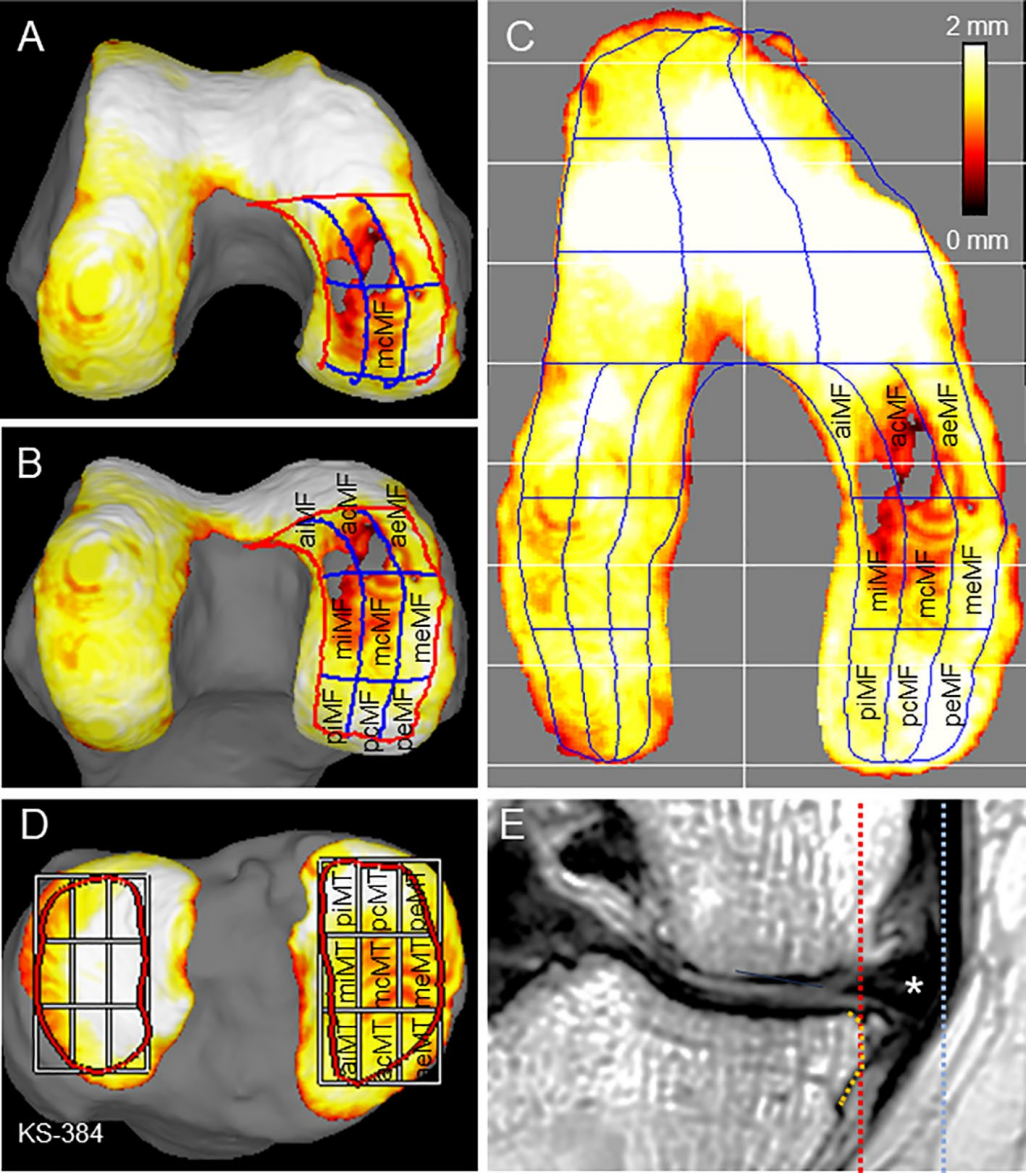
Measurement methods for subregions and medial meniscus extrusion (MME) in MRI. (**A**) Three-dimensional femoral cartilage viewed from below along the long axis of the femur. Cartilage thickness is shown with a color bar. (**B**) Three-dimensional femoral cartilage from a more posterior direction with all nine subregions visible. (**C**) Radially projected cartilage thickness mapping of the femoral cartilage and subregions of the medial femoral cartilage. The MF region is the combined area of the nine MF subregions. (**D**) Three-dimensional tibial cartilage and subregions of the medial tibial cartilage. The MT region is the combined area of the nine MT subregions. (**E**) Measurement of MME. A line along the original medial edge of the tibia (orange line) is drawn subjectively. A vertical line (red line) is drawn from the medial edge of the tibia, excluding the osteophytes, using a coronal plane MRI. A line (blue line) is then drawn from the outer edge of the medial meniscus (star). The distance between the red and blue lines is defined as the MME width. MF, medial femoral; aiMF, anterior internal MF; acMF, anterior central MF; aeMF, anterior external MF; miMF, middle internal MF; mcMF, middle central MF; meMF, middle external MF; piMF, posterior internal MF; pcMF, posterior central MF; and peMF, posterior external MF. MT, medial tibial; piMT, posterior internal MT; pcMT, posterior central MT; peMT, posterior external MT; miMT, middle internal MT; mcMT, middle central MT; meMT, middle external MT; aiMT, anterior internal MT; acMT, anterior central MT; and aeMT, anterior external MT.

The software used for MRI analyses was a 3D image analysis system volume analyzer (SYNAPSE 3D (Japanese product name: SYNAPSE VINCENT), Collaborative version 6.7, FUJIFILM Corporation, Tokyo, Japan). The bone region and region of interest (ROI) were automatically segmented from the PDW images. The cartilage region was automatically segmented from the SPGR images. The 3D image with ROI was then reconstructed. The software segmented the cartilage overlying the most superficial layer of the bone into the smallest detectable units and quantified the thickness for each unit. A visual map of the cartilage thickness was provided on a color scale, with thicker areas indicated in white and thinner areas in red. For the femoral cartilage, the ROI was automatically divided into three regions—the medial, lateral, and trochlear femoral—based on the shape of the ROI. Each region was then divided again into nine subregions, based on the shape of each region. In this study, only the medial compartment was analyzed. The software showed three-dimensional views of the femoral cartilage from below, along the long axis of the femur (Fig. 2A), and from a more posterior aspect, so that all nine subregions were visible (Fig. 2B). The femoral cartilage was also projected radially around the intercondylar axis (Fig. 2C). For the tibial cartilage, the ROI was also divided into nine subregions, with three equal vertical and horizontal divisions (Fig. 2D)^12^.

### Measurements of MME by MRI

Coronal cross-section images were reconstructed from the MRI data of the PDW sagittal cross-section images. The slice showing the maximum transverse diameter of the tibia was selected. A perpendicular line was drawn from the outer edge after exclusion of osteophytes of the tibia. A perpendicular line was also drawn from the outer edge of the medial meniscus. The distance between these two perpendicular lines was defined as the MME (Fig. 2E)^9^. For the inter-rater reliability, 527 subjects were selected from the Kanagawa Knee Study, and their MME widths were independently measured by two orthopedic surgeons with 4 years of experience (Y.M. and S.S.). The inter-rater reliability (intraclass correlation coefficient 2, 1) was 0.90 (95% confidence interval, 0.87–0.92)^9^.

### Statistical analysis

**Figure 3:**
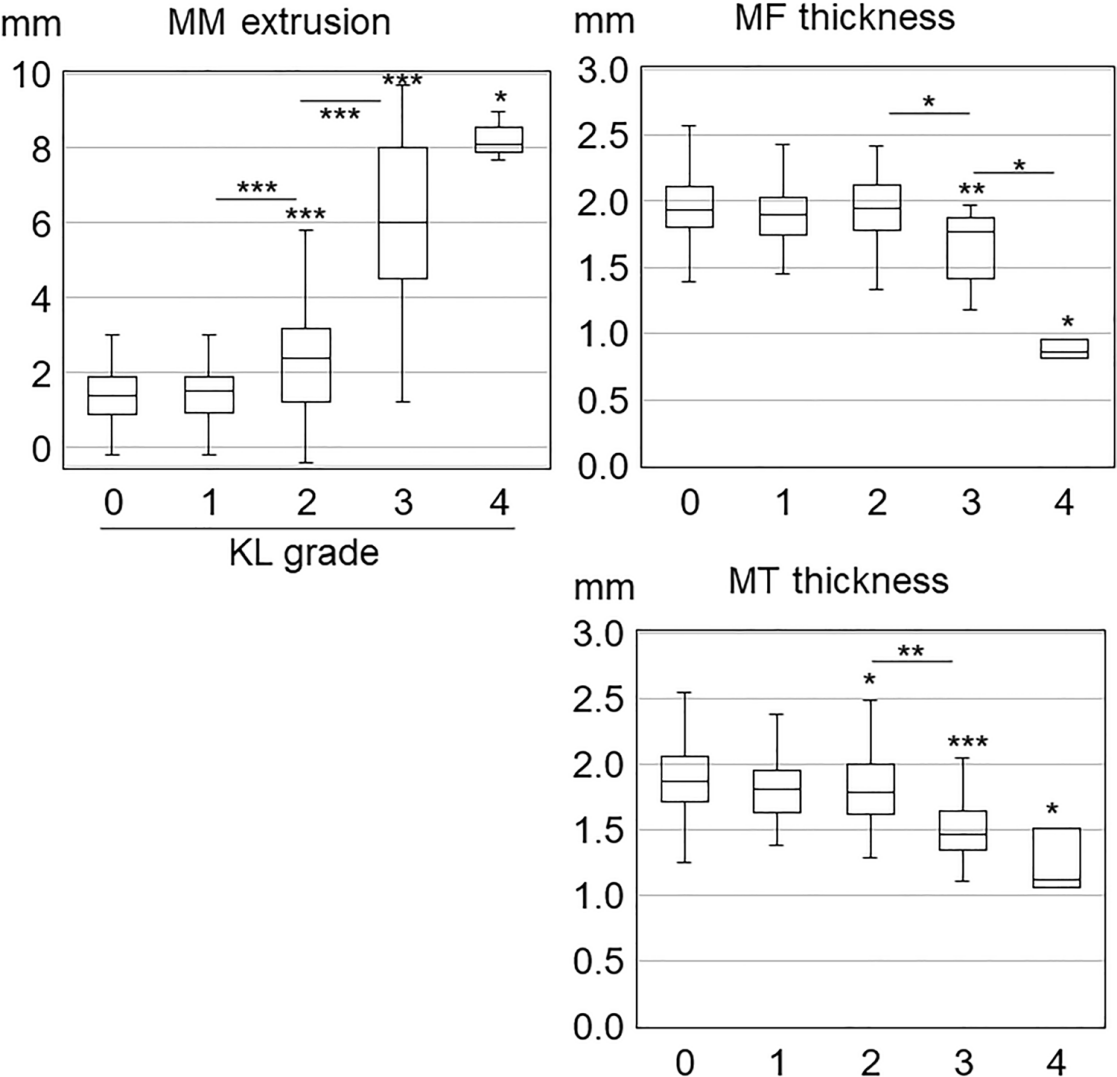
Association of Kellgren–Lawrence grade with medial meniscus extrusion width and cartilage thickness in the medial femoral and the medial tibial regions. The center line in each box plot indicates the median value, the boxed area indicates the interquartile, and the whiskers extend from either side of the box to show the minimum and maximum values (or 1.5 times the interquartile range if the minimum or maximum values fall outside this range). Asterisks over the box plots indicate p-values of multiple comparisons by the Steel–Dwass test with KL grade 0 after the Kruskal–Wallis test. Asterisks between adjacent box plots indicate the p-values of multiple comparisons by the Steel–Dwass test after the Kruskal–Wallis test. *p < 0.05, **p < 0.01, ***p < 0.001.

**Figure 4:**
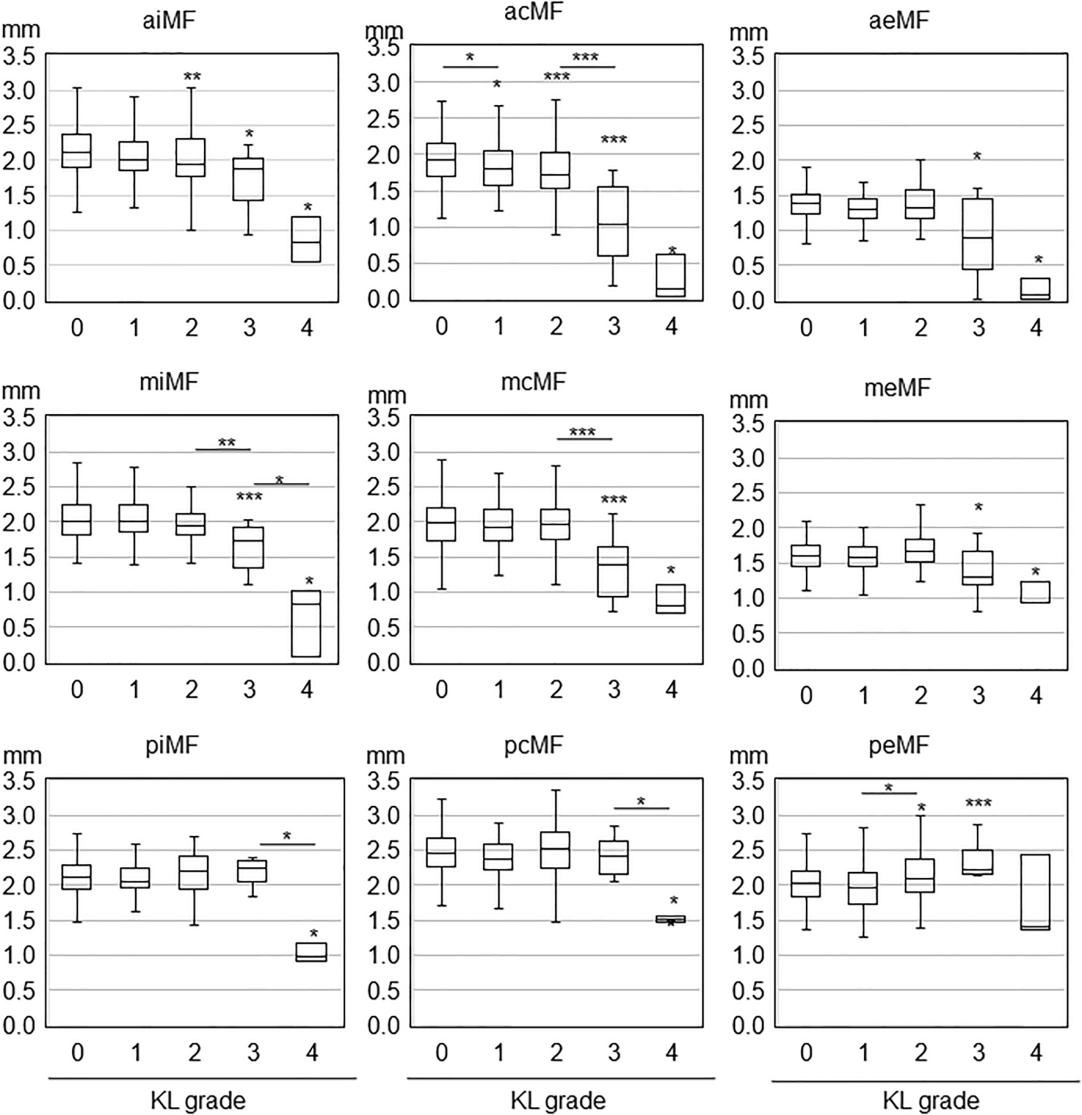
Association of Kellgren-Lawrence grade with cartilage thickness in nine medial femoral subregions. The center line in each box plot indicates the median value, the boxed area indicates the interquartile, and the whiskers extend from either side of the box to show the minimum and maximum values (or 1.5 times the interquartile range if the minimum or maximum values fall outside this range). Asterisks over the box plots indicate p-values of multiple comparisons by the Steel–Dwass test with KL grade 0 after the Kruskal–Wallis test. Asterisks between adjacent box plots indicate p-values of multiple comparisons by the Steel–Dwass test after the Kruskal–Wallis test. *; p < 0.05, **; p < 0.01, ***; p < 0.001.

**Figure 5:**
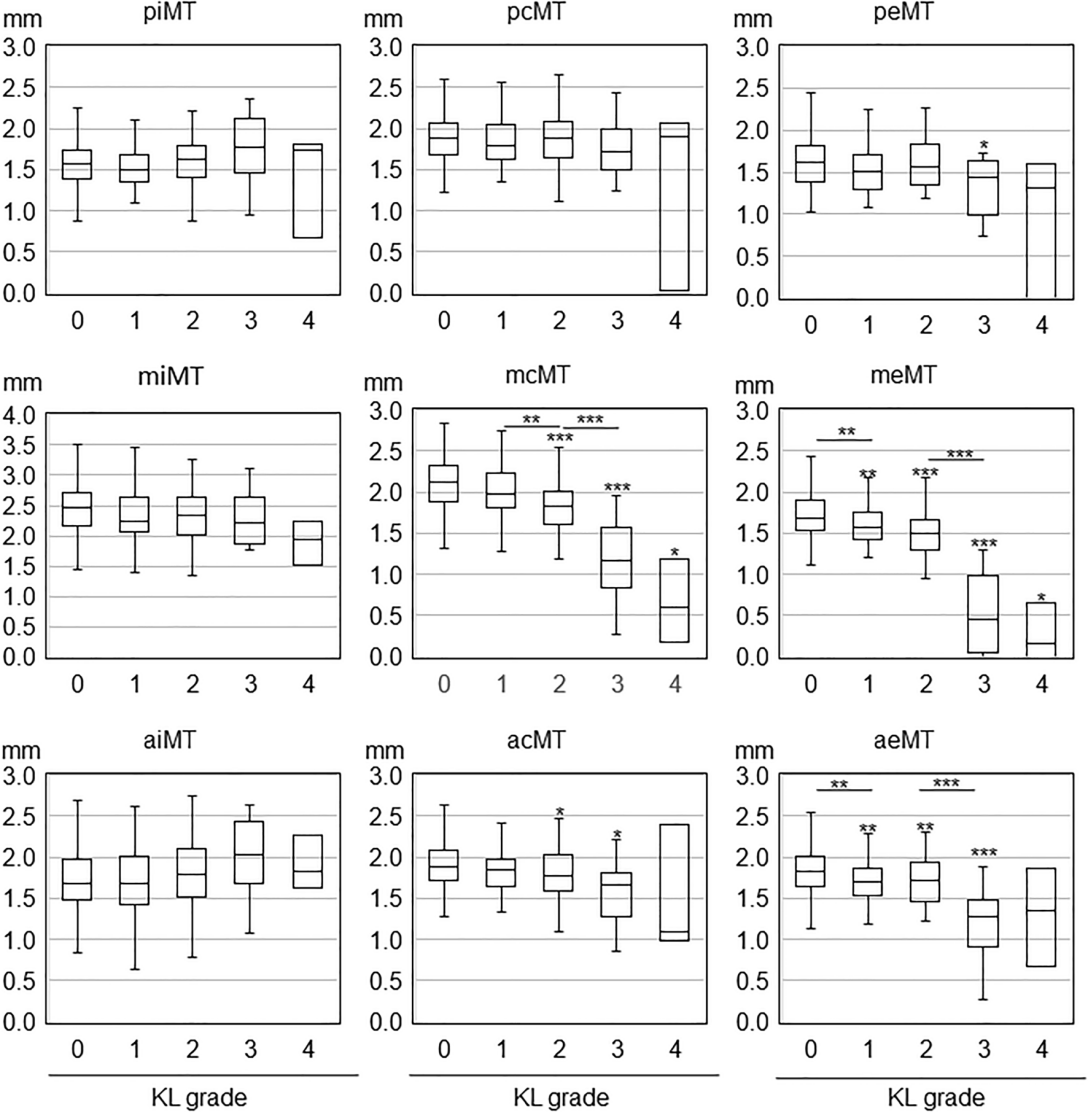
Association of Kellgren-Lawrence grade with cartilage thickness in nine medial tibial subregions. The center line in each box plot indicates the median value, the boxed area indicates the interquartile, and the whiskers extend from either side of the box to show the minimum and maximum values (or 1.5 times the interquartile range if the minimum or maximum values fall outside this range). Asterisks over the box plots indicate p-values of multiple comparisons by the Steel–Dwass test with KL grade 0 after the Kruskal–Wallis test. Asterisks between adjacent box plots indicate p-values of multiple comparisons by the Steel–Dwass test after the Kruskal–Wallis test. *p < 0.05, **p < 0.01, ***p < 0.001.

We compared the MME and cartilage thickness values in knees with KL grades of 1, 2, 3, and 4 to those in knees with KL grade 0 by multiple comparisons with the Steel–Dwass test after the Kruskal–Wallis test using the BellCurve software for Excel (Social Survey Research Information Co., Ltd. Tokyo, Japan). We also compared the values between adjacent KL grades. The results are presented as box plots (Figs. 3, 4, 5). The center line in each plot indicates the median value, the boxed area indicates the interquartile, and the whiskers extend from either side of the box to show the minimum and maximum values (or 1.5 times the interquartile range if the minimum or maximum values fall outside this range). Tests of the independence of gender differences by KL grade were performed with the chi-square test, and samples with values less than 5 were excluded. The association between MME width and cartilage thickness by KL grade was determined by correlation analysis of MME and cartilage thickness (2 regions and 18 subregions, 20 total) in each of the KL grades 0–3.

### Ethics approval and consent to participate

This study was approved by the Medical Research Ethics Committee of Tokyo Medical and Dental University, and written informed consent was obtained from all subjects. The protocol was enrolled in a database of the National University Hospital Council of Japan (UMIN000032826) and disclosed.

## Results

### Subject demographics

We enrolled 573 people. Eleven withdrew, and two had ineligible data, leaving 560 (Supplementary Fig. 1). One was excluded due to improper radiographs, and 90 were excluded due to lateral knee OA, leaving a total of 469 subjects for the current analysis.

The 469 subjects consisted of 227 (48%) women and 242 (52%) men (Table 1). The median age was 54 years (range: 30–79) and the median BMI was 22.7 kg/m^2^ (range:16.3–30.7). By KL grade, the subjects included 308 KL grade 0, 70 KL grade 1, 75 KL grade 2. 13 KL grade 3, and 3 KL grade 4 patients. Age increased significantly with an increase in KL, and BMI was significantly higher in KL grade 1 and KL grade 2 than in KL grade 0, and in KL grade 3 than in KL grade 1. The comparisons of KL grade 0 vs. KL grade 4 are based on only three subjects with KL grade 4; therefore, those results are not detailed below.

**Table 1:** Demographic of the subjects.

KL grade	P value counterpart	0	1		2			3			4			(Overall)	Overall
				KL0		KL0	KL1		KL0	KL1		KL0	KL1		
Subject	Number	308	70		75			13			3			469	
	Ratio	0.66	0.15		0.16			0.03			0.01			1.00	
Gender	Female	139	32		45			8			3			227	NS^#^
	Male	169	38		30			5			0			242	
	Female ratio	0.45	0.46		0.60			0.62			1.00			0.48	
Age	Median	49	59.5	**	66	***	**	71	***	*	75	*	*	54	***
	Maximum	79	79		78			76			79			79	
	Minimum	30	30		35			51			75			30	
BMI	Median	22.4	22.1		23.6	*		24.9	**	**	22.5			22.7	***
	Maximum	41.9	31.4		29.7			30.7			24.6			30.7	
	Minimum	16.3	16.3		17.3			22.5			22.1			16.3	

*P < 0.05, **P < 0.01, ***P < 0.001, NS: not significant.

^#^KL4 was excluded from the test because the sample size was less than 5.

There were no significant differences between the two pairs of KL2, KL3, and KL4.

### MME, medial femoral region, and medial tibial region

The median MME width was 1.4 mm in KL grade 0, and it remained statistically unchanged at 1.5 mm in KL grade 1 (normal < 2 mm), but it increased significantly to 2.4 mm in KL grade 2 (mild extrusion > 2 to 4 mm) (p < 0.001 compared to KL grade 1), to 6.0 mm in KL grade 3 (moderate extrusion > 4 to 6 mm) (p < 0.001 compared to KL grade 2), and to 8.1 mm in KL grade 4 (severe extrusion > 6 mm) (Fig. 3, Supplementary Table 2). The median cartilage thickness in the MF was 1.9 mm in KL grade 0, and showed no significant change in KL grade 1 and KL grade 2, but it significantly decreased to 1.8 mm in KL grade 3 (p < 0.05 compared to KL grade 2), and to 0.9 mm in KL grade 4 (p < 0.05 compared to KL grade 3). The median cartilage thickness in the MT was 1.9 mm in KL grade 0, and showed no significant change in KL grade 1, but it significantly decreased to 1.8 mm in KL grade 2 (p < 0.05 compared to KL grade 0), to 1.5 mm in KL grade 3 (p < 0.01 compared to KL grade 2), and to 1.1 mm in KL grade 4 (p < 0.05 compared to KL grade 0).

### Medial femoral subregions

In the anterior central medial femoral (acMF), the cartilage thickness in KL grades 1–4 was significantly lower than in KL grade 0 (Fig. 4, Supplementary Table 2). In the anterior internal MF (aiMF), the cartilage thickness was significantly lower in KL grades 2–4 than in KL grade 0. In the anterior external MF (aeMF), middle internal MF (miMF), middle central MF (mcMF), and middle external MF (meMF), the cartilage thickness was significantly lower in KL grades 3–4 than in KL grade 0.

### Medial tibial subregions

In the middle external medial tibial (meMT) and anterior external MT (aeMT), the cartilage thickness was smaller in KL grades 1–3 than in KL grade 0 (Fig. 5, Supplementary Table 2). In the middle central MT (mcMT) and anterior central MT (acMT), the cartilage thickness was lower in KL grades 2–3 than in KL grade 0. In the posterior external MT (peMT), the cartilage thickness was smaller in KL grade 3 than in KL grade 0.

### Association between MME width and cartilage thickness by KL grade

**Figure 6:**
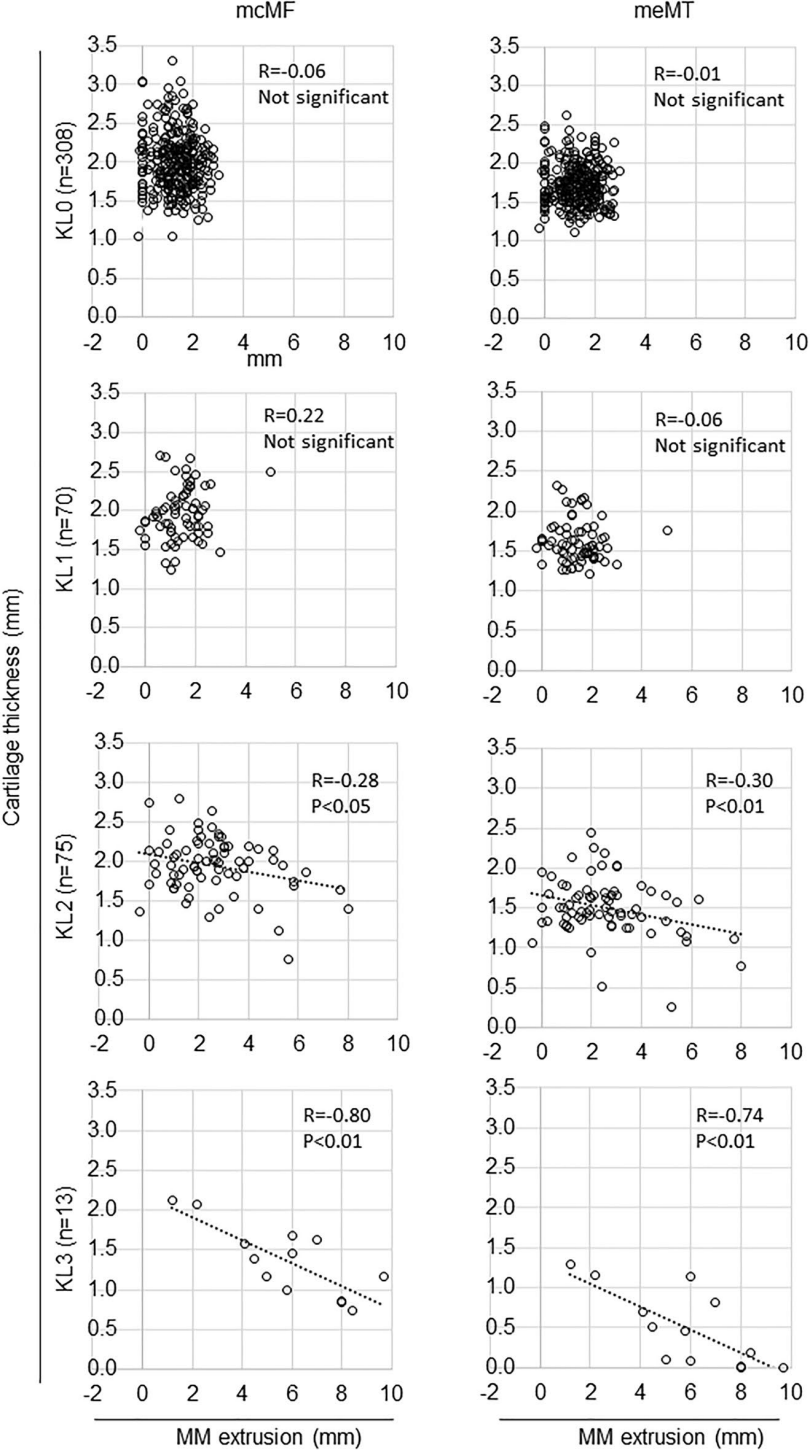
Association between MME width and cartilage thickness by KL grade in the mcMF and meMT subregions. Scatter plots between MME and cartilage thickness are shown. Regression lines are indicated when p-values are less than 0.05.

Correlation analysis of MME and cartilage thickness (2 regions and 18 subregions, 20 total) in each of the KL grades 0–3 showed significant differences in 16 of 80 analyses (Supplementary Table 3). Among them were five analyses with absolute correlation coefficients greater than 0.6, all in KL grade 3 knees. The highest absolute value of the correlation coefficient for femoral cartilage was in the mcMF, and for the tibial cartilage it was in the meMT. In both subregions, the absolute value of the correlation coefficient increased as the KL grade increased (Fig. 6).

### Summary of MME and cartilage thickness in each subregion for KL grade

**Figure 7:**
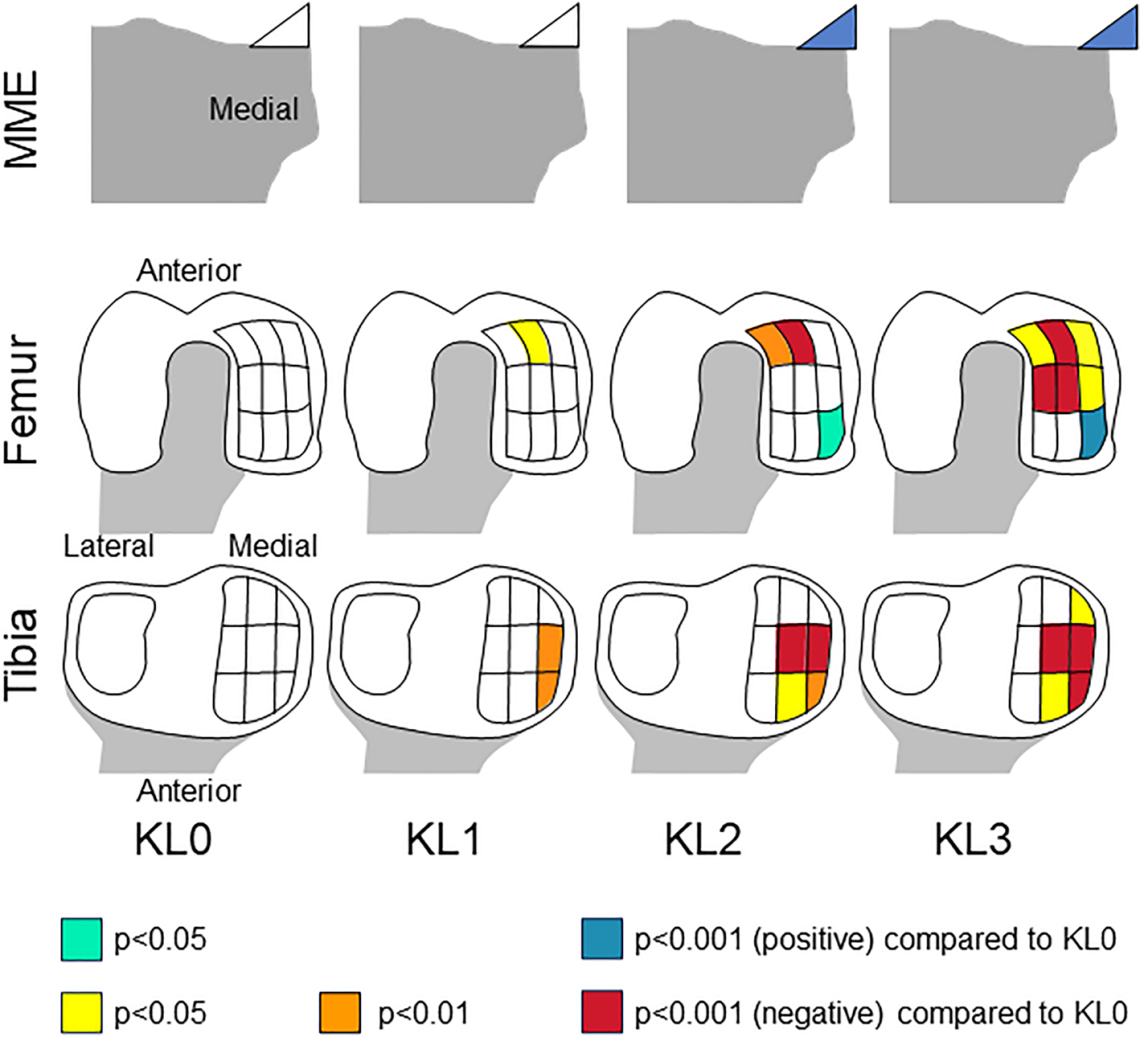
Summary of medial meniscus extrusion and cartilage thickness in each subregion for Kellgren-Lawrence grades 1–3 compared to KL grade 0. Medial meniscus and cartilage subregions are color-coded according to p-values. The MME width was similar to KL grade 0 in KL grade 1, but greater than KL grade 0 in KL grade 2 and KL grade 3; it was significantly greater in KL grade 3 than in KL grade 2. The femoral cartilage was significantly thinner in the acMF in KL grade 1, further in the aiMF in KL grade 2, and more so in four surrounding subregions in KL grade 3 when compared to KL grade 0. Conversely, the femoral cartilage was thicker in the peMF in KL grade 2 and 3 than in KL grade 0. The tibial cartilage was significantly thinner in the meMT and aeMT in KL grade 1, more so in the mcMT and acMT in KL grade 2, and even more so in peMT in KL grade 3 when compared to KL grade 0.

The MME width was similar to KL grade 0 in KL grade 1, but it was greater than KL grade 0 in KL grade 2 and KL grade 3; it was significantly greater in KL grade 3 than in KL grade 2 (Fig. 7). The femoral cartilage was significantly thinner in the acMF in KL grade 1, further in the aiMF in KL grade 2, and more so in the four surrounding subregions in KL grade 3 when compared to KL grade 0. Conversely, the femoral cartilage was thicker in the peMF in KL grade 2 and 3 than in KL grade 0. The tibial cartilage was significantly thinner in the meMT and aeMT in KL grade 1, and the difference was larger between the mcMT and acMT in KL grade 2, and more so in peMT in KL grade 3 when compared to KL grade 0.

## Discussion

We determined the relationship between KL grade and MME and the anatomical relationship between KL grade and cartilage thickness in both early and moderate OA. The MME width was unchanged in KL grade 1, slightly increased in KL grade 2, and moderately increased in KL grade 3. Cartilage thinning in the medial femur was first noted in the anterior central subregion in KL grade 1, then it expanded inwardly in KL grade 2, and further expanded externally and into the middle subregions in KL grade 3. Cartilage thinning in the medial tibia first occurred in the anterior and middle external subregions in KL grade 1, expanded into the anterior and middle central subregion in KL grade 2, and expanded further into the posterior external subregion in KL grade 3.

The MME remained unchanged in KL grade 1, and the meniscal extrusion worsened from KL grade 2 and the grades thereafter. A positive correlation between MME and tibial osteophyte width on radiographs^9^ and MRI^7^ has been previously reported, as has a correlation between the KL grade and MME^21^. However, whether the MME differed between KL grade 0 and KL grade 1, or between KL grade 1 and KL grade 2 was not entirely evident, due to the low repeatability of KL evaluations in early to moderate OA^19^.

Whether the osteophytes or the MME occurs first in OA is unclear. The lack of a difference in MME between KL grades 0 and 1 in our study could suggest that osteophytes occur first. However, it is important to consider that all MRI scans in this study were conducted in the supine position, which could affect the measurement of MME, as MME has been reported to increase during weight-bearing scenarios^22^. This implies that the MRI-based measurements of MME in this study may be biased toward lower values than would be observed in a weight-bearing position. Similarly, cartilage thickness is known to decrease during weight-bearing^23^, particularly in central regions^24^. Therefore, the measurements of MME and cartilage thickness using MRI in this study could be influenced by the use of non-weight-bearing positions, lending further support for evaluation in the context of the potential bias introduced by the lack of weight-bearing, based on previous research. Consequently, due to the possible confounding effects of weight-bearing positioning, this study did not definitively determine whether osteophytes or MME occur first in OA.

Cartilage thinning in the medial tibia was first observed in the anterior and middle external subregions in KL grade 1, and it was subsequently observed in the surrounding areas. Our previous 3D MRI analysis of subjects with cartilage defects on the medial tibia revealed the presence of cartilage defects in an area that extended from the middle external subregion to the periphery when arranged according to the defect size^8^. Conversely, unlike those previous findings, thinning was observed in the present study in the anterior external subregion, as well as in the middle external subregion, in KL grade 1. This difference is important because KL grade 1 is considered to represent pre-radiographic OA and is not supposed to show any cartilage damage.

The femoral cartilage thinning initiated from the anterior central subregion and spread around the periphery. The anterior and middle subregions can be seen when viewed from below along the long axis of the femur, suggesting that these subregions are the areas where contact occurs during knee extension in the standing position and could explain the thinning of these subregions observed in KL grade 3. By contrast, the lack of thinning in the posterior subregions up to KL grade 3 and the thickening of cartilage in the posterior external subregion could reflect less loading on the posterior subregions^25^.

The observed differences in cartilage thickness between KL grade 0 and KL grade 1 are clinically relevant because they provide insight into the early stages of knee joint changes. The thinning of cartilage in KL grade 1 suggests that the disease process has already begun, and this could have implications for disease progression and treatment. In a clinical context, these findings could influence decisions regarding interventions and patient education. For example, individuals with KL grade 1 who may have unequivocal radiographic changes could still benefit from early interventions aimed at preserving joint function and preventing further progression of cartilage loss. These interventions could include recommendations for lifestyle changes, targeted exercise, and patient education about joint health^26^.

The association between MME width and cartilage thickness by KL grade in meMT revealed no association with KL grades 0 and 1, but a significant correlation with KL grade 2, and a stronger correlation with KL grade 3. KL grade 2 is characterized by unequivocal osteophytes, which increase the range of associated MME^26^, and this would reveal an association between MME width and cartilage thickness. KL grade 3 is characterized by joint space narrowing, which increases the range of cartilage thickness, and this would reveal a stronger association between MME width and cartilage thickness. A similar explanation could be proposed for mcMF.

The areas of cartilage thinning in each KL in this study suggested the following mechanism for OA progression, based on three assumptions: the tibial cartilage that is no longer covered by the meniscus is easily worn away^27^; femoral cartilage wears away in the area that contacts the area of tibial cartilage thinning^28^; and lateral thrust is an aggravating factor in medial OA^29^. For KL grade 1, MRI shows no MME, and this causes thinning of the aeMT and meMT. The acMF of this mirror lesion is also thinned. KL grade 2 shows a mild MME, and the aeMT and the other three subregions adjacent to the aeMT are thinned. In addition, femoral cartilage thinning occurs in the aiMF rather than the aeMF due to a lateral thrust. For KL grade 3, the MME becomes moderate, and the tibial cartilage thins to the peMT. The femoral cartilage thins in the six anterior and middle subregions, which are the mirror lesions of these tibial lesions. The three posterior regions are less affected by loading and do not thin, whereas the peMF thickens.

Cartilage thickness changes were analyzed by White et al.^30^, who performed a gross analysis of 46 cases of tibial cartilage resected during unilateral knee OA arthroplasty. Assessment of the medial central tibial cartilage in the center from anterior to posterior revealed a decrease in cartilage thickness in the anterior and middle tibial cartilage^30^. This previous study is considered to have targeted KL grades 3–4, and our results are consistent with KL grade 3, as the cartilage thickness was reduced in acMT and mcMT. Reichenbach et al.^31^ analyzed the relationship between KL grade and cartilage thickness in 948 knees from the Framingham OA Study. In that study, the subregions corresponding to our definition of mcMF showed significantly thinner cartilage thickness in KL 2–3 and 4, and the subregion corresponding to mcMT showed significantly thinner cartilage thickness in KL 2–3 in females and in KL 2–4 in males, compared to KL 0. These findings are consistent with our KL grades 3–4, but in some respects, they are inconsistent with KL grade 2. This study differed from ours in that the MRI was taken with 3D FLASH-water excitation sequences, and the segmentation was done manually and analyzed separately for females and males. Guillard et al.^32^ performed 3D MRI analysis of 8890 knees from the Osteoarthritis Initiative to quantify cartilage thickness in the central medial femur and central medial tibia for each KL grade. Cartilage thickness was unchanged between KL grade 0 and KL grade 2, and it decreased gradually in KL grades 3 and 4. These regions corresponded to our mcMF and mcMT subregions, and their results are consistent with our results for mcMF. The advantage of our study, compared to these previous studies, is that we analyzed the surrounding 8 subregions for the femur and tibia in addition to the central subregions, and we added the MME to our analyses.

KL grading was done automatically using KOALA AI software. Rough checks were performed by human eyes, checking for laterality errors due to poor x-ray conditions, but we prevented bias by making no manual changes. Three previous reports have evaluated the accuracy of KOALA software. Nehrer et al.^4^ analyzed 124 knee radiographs from the Osteoarthritis Initiative study and reported a 21% increase in KL grade concordance when KOALA software was used than when the software was not used. Smolle et al.^33^ reported that the concordance rate for KL grades by three certified orthopedic surgeons for 124 knee radiographs was twice as high with KOALA software than without KOALA software. Neubauer et al.^34^ analyzed 69 subjects in the MLKOA physical therapy trial and reported an increase in KL grade concordance when KOALA software was used than when KOALA software was not used. The greatest problem with KL grading is the high variability among raters, but this problem can be reduced with the use of automatic KL grading software, such as KOALA software, especially when the readers are not musculoskeletal radiologists or when they lack extensive experience in clinical trial readings.

In the present study, knee radiographs were taken in extension, with the knees in the upright position. This was done to follow the original method for KL grading. Kothari et al.^35^ reported that a mild knee flexion position contributes to reproducible joint space width. In fact, KOALA software uses radiographs taken at mild flexion using the Synaflexer as training data. While we have empirically confirmed that KOALA software can classify the KL grade from radiographs with knee extension without notable issues, one limitation to report is that the inter-image error is higher for radiographs taken with knee extension than for radiographs acquired with mild knee flexion.

We have reported on the accuracy of 3D MRI analysis by SYNAPSE 3D in two previous papers. First, we ran a validation test for our algorithm by randomly selecting 108 of the 113 subjects for training, and the other 5 subjects were used for a validation test by computing the Dice similarity coefficient (DSC)^36^. The mean DSC was 0.99 for the femoral bone, 0.98 for the tibial bone, 0.91 for the femoral cartilage, and 0.89 for the tibial cartilage^17^. A DSC of 1 indicates a perfect match, and 0 indicates a perfect mismatch. Our values were over 0.98 for bone and over 0.89 for cartilage, indicating a high degree of matching. Second, nine healthy volunteers underwent MRI twice in the same day, and the interscan measurement error (the absolute difference obtained by subtracting the second MRI from the first MRI) was calculated at each region and subregion from the data. The interscan measurement error for the cartilage thickness in the medial femoral region was 0.03 ± 0.02 mm (mean ± standard deviation), and the interscan measurement error for the nine subregions of the medial femoral cartilage region ranged from 0.04 ± 0.02 mm to 0.11 ± 0.10 mm. The interscan measurement error for the cartilage thickness in the medial tibial region was 0.03 ± 0.02 mm, and the interscan measurement error of the nine subregions of the medial femoral cartilage region ranged from 0.04 ± 0.03 mm to 0.11 ± 0.07 mm^9^. These interscan measurement errors seem to be sufficient to conduct this study.

The slice showing the maximum transverse diameter of the tibia in the coronal plane was selected for the MME measurements. However, this method may not always be accurate, as the shape of the tibia can vary from person to person, and the maximum transverse diameter may not always show the maximum MME width. Three-dimensional imaging can improve accuracy and reduce limitations in the MME measurements by providing a more complete view of the MM and the tibia.

The subjects in this study were significantly older as the KL grade increased, and the BMI was significantly higher for KL grades 2–3 than for KL grade 0. Both the MME^37^ and the cartilage thickness loss^38^ are associated with age and BMI; therefore, age and BMI should be considered potential confounding factors. However, we did not adjust for these factors in our analysis due to the decreasing number of subjects across the increasing KL grades, which culminated in a very small sample size for KL grade 3. Taking this limitation into account, future investigations are encouraged to control for age and BMI to deepen our understanding of their roles in the association between KL grade, MME, and cartilage thickness reduction.

Tibiofemoral knee OA comprises medial and lateral tibiofemoral OA; however, only medial OA was analyzed in the present study. The main reason for this limitation was that the anatomy, kinematics, and loading during movement differ considerably between the medial and lateral knee compartments of the knee^39^. Therefore, we were concerned that the analysis would be too complicated if we included both compartments. Furthermore, medial OA occurs more commonly than lateral OA^39^, and medial OA is more likely to result in surgery^40^. The medial compartment is also the focus of disease-modifying osteoarthritis disease (DMOAD) clinical trials. For these reasons, lateral OA was excluded from the present study, and therefore, the findings of this study cannot be generalized to patients with lateral tibiofemoral knee OA.

Our study has several other limitations. First, the KL grade was determined using an automated grading system to improve repeatability. However, this would not necessarily be consistent with expert consensus. Second, because we used an automated MRI analysis system that cannot yet measure MME, we had to measure the MME manually. The inter-rater repeatability of each measurement is reliable^9^, but the potential for measurement errors cannot be ignored. Third, among the 469 subjects included in this study, only 3 were graded as KL grade 4. We included the results for KL grade 4, but we excluded this grade from the discussion. Fourth, the subjects analyzed in this study are not representative of the general population; therefore, the results of the study must be interpreted with caution before they can be generalized.

We propose three future studies. First, our evaluation of MME used only a single slice from the MRI coronal sequence; therefore, we may not have captured the entire MME pathology. MME can potentially move maximally in the anterior, anteromedial, and posteromedial directions; hence, the next step should be to perform a more detailed analysis by assessing the direction of MME in two dimensions. Second, because the current study focused on individuals with no history of knee symptoms, KL grades 0–2 accounted for 97% of the cohort. In the next study, we will focus on groups with a high percentage of KL grades 3–4. Third, this analysis used cross-sectional data from the Kanagawa Knee Study, although this study also has longitudinal data collected at one-year intervals. The next step would be to use this longitudinal data set to further analyze the relationship between KL grades and cartilage thickness loss/ MME progression.

## Conclusion

MME was unchanged in KL grade 1 and KL grade 0, whereas it increased slightly in KL grade 2 and increased moderately in KL grade 3. Cartilage thickness first decreased in the medial tibial region in KL grade 2 subjects and in the medial femoral region in KL grade 3. Cartilage thinning occurred in the anterior central medial femoral subregion and in the anterior and middle external medial tibial subregions in KL grade 1, whereas it diffusely expanded in early and moderate knee OA. The findings of this study provide novel insights into the early stages of knee OA and its disease progression and potentially have implications for the development of early intervention strategies and for the diagnosis and management of knee OA.

### Supplementary Information

Supplementary Table 1.

Supplementary Table 2.

Supplementary Table 3.

Supplementary Figure 1.

## Data Availability

The data sets used and/or analyzed during the current study are available from the corresponding author on reasonable request.

## Abbreviations

AI: Artificial intelligence
MRI: Magnetic resonance imaging
3D MRI: Three-dimensional MRI
2D MRI: Two-dimensional MRI
OA: Osteoarthritis
MM: Medial meniscus
MME: MM extrusion
SPGR: Spoiled gradient
PDW: Proton-density weighted
KL: Kellgren–Lawrence
DICOM: Digital imaging and communications in medicine
