# Supplementary material for: Association of AI-determined Kellgren–Lawrence grade with medial meniscus extrusion and cartilage thickness by AI-based 3D MRI analysis in early knee osteoarthritis
Source: Sci Rep. 2023 Nov 16;13:20093. doi: 10.1038/s41598-023-46953-9 (PMC10654518; doi:10.1038/s41598-023-46953-9)
Supplement: Supplementary file 1 — Supplementary Table 1. [file 41598_2023_46953_MOESM1_ESM.docx]

| Supplementary Table 1. Imaging Parameters for the MRI Sequences. | | |
| --- | --- | --- |
|  | SPGR | PDW |
| Repetition time (msec) | 20 | 1000 |
| Echo time (msec) | 1st: 7 | 35 |
|  | 2nd: 13.8 |  |
| Flip angles (°) | 35 | 90 |
| Echo train length | (−) | 32 |
| Acquisition voxel size (mm) | 0.6 × 0.6 × 0.6 | 0.6 × 0.6 × 0.6 |
| Reconstruction matrix size (mm) | 0.3 × 0.3 × 0.3 | 0.3 × 0.3 × 0.3 |
| No. of slices | 320 | 320 |
| Slice thickness (mm) | 0.3 | 0.3 |
| Slice gap (mm) | 0 | 0 |
| Field of view (mm × mm) | 150 × 150 | 150 × 150 |
| WFS/BW (pix/Hz) | 2.002/217.0 | 0.836/519.4 |
| Number of excitations | 1 | 1 |
| Total examination time | 7 min 30 sec | 7 min 10 sec |
| SPGR = fat-suppressed spoiled gradient echo; PDW = proton density weighted; WFS/BW = actual waterfat shift/bandwidth; MRI = magnetic resonance imaging. | | |
