# Supplementary material for: Association of AI-determined Kellgren–Lawrence grade with medial meniscus extrusion and cartilage thickness by AI-based 3D MRI analysis in early knee osteoarthritis
Source: Sci Rep. 2023 Nov 16;13:20093. doi: 10.1038/s41598-023-46953-9 (PMC10654518; doi:10.1038/s41598-023-46953-9)
Supplement: Supplementary file 2 — Supplementary Table 2. [file 41598_2023_46953_MOESM2_ESM.docx]

| Supplementary Tabe 2. Measured values of MME and cartilage thickness in each region and subregion. | | | | | | | | | | | | | | | | | | | | | | |
| --- | --- | --- | --- | --- | --- | --- | --- | --- | --- | --- | --- | --- | --- | --- | --- | --- | --- | --- | --- | --- | --- | --- |
| **KL grade** | **0** |  | **1** |  |  | **2** |  |  |  | **3** |  |  |  |  |  |  | **4** |  |  |  |  |  |
| P value counterpart |  |  |  |  | KL0 |  |  | KL0 | KL1 |  |  |  |  | KL0 | KL1 | KL2 |  |  | KL0 | KL1 | KL2 | KL3 |
| MME | 1.4 | (-0.2 - 3.0) | 1.5 | (-0.2 - 5.0) |  | 2.4 | (-0.4 - 8.0) | *** | *** | 6.0 | (1.2 - 9.7) | 1.2 | 9.7 | *** | *** | *** | 8.1 | (7.7 - 9.0) | * | * | * |  |
| MF | 1.9 | (1.2 - 2.7) | 1.9 | (1.5 - 2.4) |  | 2.0 | (1.2 - 2.7) |  |  | 1.8 | (1.2 - 2.0) | 1.2 | 2.0 | ** | * | * | 0.9 | (0.8 - 1.0) | * | * | * | * |
| peMF | 2.0 | (1.2 - 3.2) | 2.0 | (1.3 - 2.8) |  | 2.1 | (1.1 - 3.1) | * | * | 2.2 | (2.1 - 2.9) | 2.1 | 2.9 | *** | *** |  | 1.4 | (1.4 - 2.4) |  |  |  |  |
| meMF | 1.6 | (1.0 - 2.5) | 1.6 | (1.1 - 2.2) |  | 1.7 | (1.2 - 2.3) |  |  | 1.3 | (0.8 - 1.9) | 0.8 | 1.9 | * |  |  | 0.9 | (0.9 - 1.2) | * | * | * |  |
| aeMF | 1.4 | (0.7 - 2.0) | 1.3 | (0.8 - 1.9) |  | 1.3 | (0.3 - 2.0) |  |  | 0.9 | (0.0 - 1.6) | 0.0 | 1.6 | * |  |  | 0.1 | (0.0 - 0.3) | * | * | * |  |
| pcMF | 2.5 | (1.6 - 3.5) | 2.4 | (1.7 - 2.9) |  | 2.5 | (1.2 - 3.4) |  |  | 2.4 | (2.1 - 2.8) | 2.1 | 2.8 |  |  |  | 1.5 | (1.5 - 1.6) | * | * | * | * |
| mcMF | 2.0 | (1.1 - 3.3) | 1.9 | (1.2 - 2.7) |  | 2.0 | (0.8 - 2.8) |  |  | 1.4 | (0.7 - 2.1) | 0.7 | 2.1 | *** | *** | *** | 0.8 | (0.7 - 1.1) | * | * | * |  |
| acMF | 1.9 | (1.0 - 2.9) | 1.8 | (1.2 - 2.7) | * | 1.7 | (0.4 - 3.0) | *** |  | 1.0 | (0.2 - 1.8) | 0.2 | 1.8 | *** | *** | *** | 0.2 | (0.0 - 0.6) | * | * | * |  |
| piMF | 2.1 | (1.3 - 3.0) | 2.1 | (1.3 - 2.6) |  | 2.2 | (1.4 - 2.7) |  |  | 2.3 | (1.8 - 2.9) | 1.8 | 2.9 |  |  |  | 1.0 | (0.9 - 1.2) | * | * | * | * |
| miMF | 2.0 | (1.1 - 3.3) | 2.0 | (1.4 - 3.0) |  | 2.0 | (0.9 - 2.8) |  |  | 1.7 | (1.1 - 2.0) | 1.1 | 2.0 | *** | ** | ** | 0.8 | (0.1 - 1.0) | * | * | * | * |
| aiMF | 2.1 | (1.3 - 3.6) | 2.0 | (1.3 - 3.0) |  | 1.9 | (1.0 - 3.4) | ** |  | 1.9 | (0.9 - 2.2) | 0.9 | 2.2 | * |  |  | 0.8 | (0.6 - 1.2) | * | * | * |  |
| MT | 1.9 | (1.2 - 2.6) | 1.8 | (1.4 - 2.5) |  | 1.8 | (1.3 - 2.5) | * |  | 1.5 | (1.1 - 2.0) | 1.1 | 2.0 | *** | *** | ** | 1.1 | (1.1 - 1.5) | * | * | * |  |
| peMT | 1.6 | (1.0 - 2.6) | 1.5 | (1.1 - 2.6) |  | 1.6 | (0.3 - 2.3) |  |  | 1.4 | (0.7 - 1.7) | 0.7 | 1.7 | * |  |  | 1.3 | (0.0 - 1.6) |  |  |  |  |
| meMT | 1.7 | (1.1 - 2.6) | 1.6 | (1.2 - 2.3) | ** | 1.5 | (0.3 - 2.4) | *** |  | 0.5 | (0.0 - 1.3) | 0.0 | 1.3 | *** | *** | *** | 0.2 | (0.0 - 0.7) | * | * | * |  |
| aeMT | 1.8 | (1.1 - 2.9) | 1.7 | (1.2 - 2.5) | ** | 1.7 | (1.2 - 2.8) | ** |  | 1.3 | (0.3 - 1.9) | 0.3 | 1.9 | *** | *** | *** | 1.4 | (0.7 - 1.9) |  |  |  |  |
| pcMT | 1.9 | (1.2 - 2.7) | 1.8 | (1.4 - 2.7) |  | 1.9 | (1.1 - 2.7) |  |  | 1.7 | (1.2 - 2.4) | 1.2 | 2.4 |  |  |  | 1.9 | (0.0 - 2.1) |  |  |  |  |
| mcMT | 2.1 | (1.3 - 3.1) | 2.0 | (1.3 - 3.2) |  | 1.8 | (0.8 - 2.9) | *** | ** | 1.2 | (0.3 - 2.0) | 0.3 | 2.0 | *** | *** | *** | 0.6 | (0.2 - 1.2) | * | * | * |  |
| acMT | 1.9 | (1.3 - 2.7) | 1.8 | (1.3 - 2.4) |  | 1.8 | (1.1 - 3.0) | * |  | 1.7 | (0.1 - 2.2) | 0.1 | 2.2 | * |  |  | 1.1 | (1.0 - 2.4) |  |  |  |  |
| piMT | 1.6 | (0.9 - 2.5) | 1.5 | (1.1 - 2.3) |  | 1.6 | (0.9 - 2.7) |  |  | 1.8 | (1.0 - 2.4) | 1.0 | 2.4 |  |  |  | 1.7 | (0.7 - 1.8) |  |  |  |  |
| miMT | 2.5 | (1.2 - 3.8) | 2.3 | (1.4 - 3.5) |  | 2.3 | (1.3 - 3.3) |  |  | 2.2 | (1.8 - 3.1) | 1.8 | 3.1 |  |  |  | 1.9 | (1.5 - 2.2) |  |  |  |  |
| aiMT | 1.7 | (0.7 - 2.9) | 1.7 | (0.6 - 2.6) |  | 1.8 | (0.8 - 2.7) |  |  | 2.0 | (1.1 - 2.6) | 1.1 | 2.6 |  |  |  | 1.8 | (1.6 - 2.3) |  |  |  |  |
| Median (Minimum - Maximum) mm. | | | | | | | | | | | | | | | | | | | | | | |
| *：P<0.05, **：P<0.01, ***：P<0.001. | | | | | | | | | | | | | | | | | | | | | | |
