# Supplementary material for: Association of AI-determined Kellgren–Lawrence grade with medial meniscus extrusion and cartilage thickness by AI-based 3D MRI analysis in early knee osteoarthritis
Source: Sci Rep. 2023 Nov 16;13:20093. doi: 10.1038/s41598-023-46953-9 (PMC10654518; doi:10.1038/s41598-023-46953-9)
Supplement: Supplementary file 3 — Supplementary Table 3. [file 41598_2023_46953_MOESM3_ESM.docx]

| Supplementary Table 3. Correlation analysis between MME width and cartilage thickness in each region and subregion by KL grade. | | | | | | | | |
| --- | --- | --- | --- | --- | --- | --- | --- | --- |
|  | P value |  |  |  | Correlation coefficients. | | |  |
|  | KL0 | KL1 | KL2 | KL3 | KL0 | KL1 | KL2 | KL3 |
| MF |  | ** |  | * | -0.01 | 0.34 | -0.16 | -0.61 |
| peMF |  |  |  |  | 0.05 | 0.17 | 0.00 | -0.14 |
| meMF |  | *** |  |  | 0.06 | 0.40 | 0.02 | -0.49 |
| aeMF | ** | ** |  |  | 0.15 | 0.37 | 0.05 | -0.37 |
| pcMF |  |  | * |  | -0.06 | 0.18 | -0.26 | -0.44 |
| mcMF |  |  | * | ** | -0.06 | 0.22 | -0.28 | -0.80 |
| acMF |  | * |  |  | 0.06 | 0.28 | -0.18 | -0.48 |
| piMF |  |  |  |  | -0.03 | 0.19 | -0.09 | -0.30 |
| miMF | * |  |  | ** | -0.14 | 0.15 | -0.20 | -0.77 |
| aiMF |  |  |  |  | -0.03 | 0.22 | -0.05 | -0.11 |
| MT |  |  |  |  | 0.01 | 0.04 | 0.04 | -0.33 |
| peMT |  |  |  |  | -0.01 | 0.01 | -0.13 | -0.29 |
| meMT |  |  | ** | ** | -0.01 | -0.06 | -0.30 | -0.74 |
| aeMT |  |  |  |  | -0.04 | 0.12 | 0.13 | -0.38 |
| pcMT |  |  |  |  | 0.05 | 0.02 | 0.05 | -0.01 |
| mcMT |  |  |  | * | 0.00 | -0.15 | -0.22 | -0.67 |
| acMT |  |  |  |  | 0.00 | 0.19 | 0.11 | 0.06 |
| piMT |  |  | * |  | -0.02 | 0.03 | 0.26 | 0.28 |
| miMT |  |  |  |  | 0.05 | -0.01 | 0.13 | -0.12 |
| aiMT |  |  | * |  | 0.01 | 0.22 | 0.30 | 0.33 |
| *：P<0.05, **：P<0.01, ***：P<0.001. | | | | | | | | |
| Values with significant differences in the correlation coefficients are highlighted in squares. | | | | | | | | |
