# Supplementary figures and images for: Association of AI-determined Kellgren–Lawrence grade with medial meniscus extrusion and cartilage thickness by AI-based 3D MRI analysis in early knee osteoarthritis
Source: Sci Rep. 2023 Nov 16;13:20093. doi: 10.1038/s41598-023-46953-9 (PMC10654518; doi:10.1038/s41598-023-46953-9)

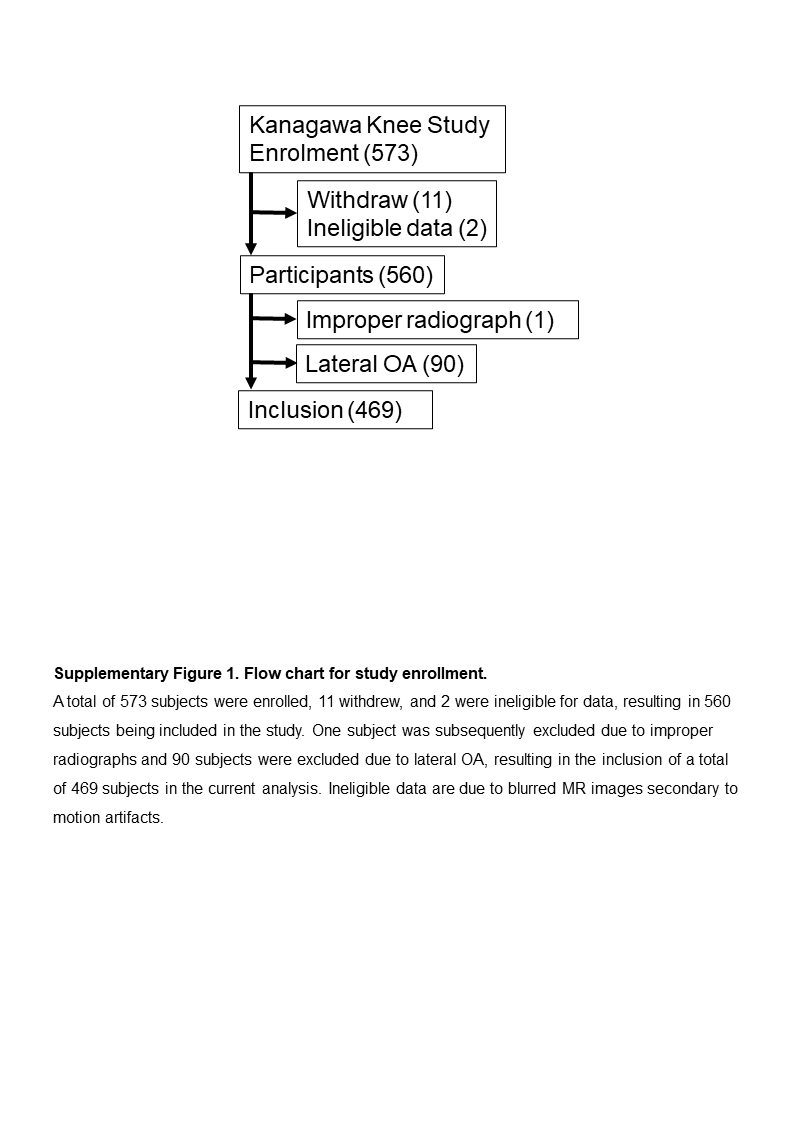

Supplement: Supplementary file 4 — Supplementary Figure 1. [file 41598_2023_46953_MOESM4_ESM.tif]
